# Adult-onset autosomal dominant spastic paraplegia linked to a GTPase-effector domain mutation of dynamin 2

**DOI:** 10.1186/s12883-015-0481-3

**Published:** 2015-10-30

**Authors:** Nyamkhishig Sambuughin, Lev G. Goldfarb, Tatiana M. Sivtseva, Tatiana K. Davydova, Vsevolod A. Vladimirtsev, Vladimir L. Osakovskiy, Al’bina P. Danilova, Raisa S. Nikitina, Anastasia N. Ylakhova, Margarita P. Diachkovskaya, Anna C. Sundborger, Neil M. Renwick, Fyodor A. Platonov, Jenny E. Hinshaw, Camilo Toro

**Affiliations:** Consortium for Health and Military Performance, Uniformed Services University, Bethesda, MD 20814 USA; National Institute of Neurological Disorders and Stroke, National Institute of Health, Bethesda, MD 20892 USA; Institute of Health, M.K. Ammosov North-Eastern Federal University, Sergelyakhskoe shosse 4 km, building C-2, Yakutsk, 677010 The Russian Federation; National Institute of Diabetes and Digestive and Kidney Diseases, National Institutes of Health, Fishers Lane, Room 4S26, Bethesda, MD 20892 USA; Department of Pathology and Molecular Medicine, Queen’s University, Kingston General Hospital, Kingston, ON K7L 3N6 Canada; National Human Genome Research Institute, National Institutes of Health, Bethesda, MD 20892 USA

**Keywords:** Paraplegia, HSP, Dynamin, *DNM2*, Neuropathy, Exome sequencing, Endocytosis

## Abstract

**Background:**

Hereditary Spastic Paraplegia (HSP) represents a large group of clinically and genetically heterogeneous disorders linked to over 70 different loci and more than 60 recognized disease-causing genes. A heightened vulnerability to disruption of various cellular processes inherent to the unique function and morphology of corticospinal neurons may account, at least in part, for the genetic heterogeneity.

**Methods:**

Whole exome sequencing was utilized to identify candidate genetic variants in a four-generation Siberian kindred that includes nine individuals showing clinical features of HSP. Segregation of candidate variants within the family yielded a disease-associated mutation. Functional as well as *in-silico* structural analyses confirmed the selected candidate variant to be causative.

**Results:**

Nine known patients had young-adult onset of bilateral slowly progressive lower-limb spasticity, weakness and hyperreflexia progressing over two-to-three decades to wheel-chair dependency. In the advanced stage of the disease, some patients also had distal wasting of lower leg muscles, *pes cavus*, mildly decreased vibratory sense in the ankles, and urinary urgency along with electrophysiological evidence of a mild distal motor/sensory axonopathy. Molecular analyses uncovered a missense c.2155C > T, p.R719W mutation in the highly conserved GTP-effector domain of dynamin 2. The mutant *DNM2* co-segregated with HSP and affected endocytosis when expressed in HeLa cells. *In-silico* modeling indicated that this HSP-associated dynamin 2 mutation is located in a highly conserved bundle-signaling element of the protein while dynamin 2 mutations associated with other disorders are located in the stalk and PH domains; p.R719W potentially disrupts dynamin 2 assembly.

**Conclusion:**

This is the first report linking a mutation in dynamin 2 to a HSP phenotype. Dynamin 2 mutations have previously been associated with other phenotypes including two forms of Charcot-Marie-Tooth neuropathy and centronuclear myopathy. These strikingly different pathogenic effects may depend on structural relationships the mutations disrupt. Awareness of this distinct association between HSP and c.2155C > T, p.R719W mutation will facilitate ascertainment of additional *DNM2* HSP families and will direct future research toward better understanding of cell biological processes involved in these partly overlapping clinical syndromes.

## Background

Hereditary spastic paraplegia (HSP) comprises a group of clinically and genetically heterogeneous diseases that affect the upper motor neurons and their long axonal projections. The hallmark of hereditary spastic paraplegia (HSP) is progressive degeneration of the corticospinal tracts, often in a length-dependent manner, and progressive spasticity as the major clinical feature; neuropathological studies have identified axon degeneration of the corticospinal tracts and fasciculus gracilis fibers [1]. The key diagnostic findings are lower limb weakness, increased muscle tone, hyperreflexia, extensor plantar responses, and gait spasticity [2].

The burden inherent to corticospinal neurons with their long axonal processes spanning from motor cortex to distant segments of the spinal cord is likely to involve various cellular vulnerabilities that could account for the broad genetic heterogeneity of HSPs. Variant forms of HSP arise from mutations in genes directly implicated in membrane traffic, mitochondrial function, endoplasmic reticulum shaping/distribution, myelination, lipid/cholesterol metabolism, bone morphogenetic protein signaling, and endolysosomal function, as well as endosome and microtubule dynamics [3, 4]. Typically, the longest fibers — those connecting to the lower spinal cord segments — are the earliest to experience changes in their terminal regions that predate changes in cell bodies, leading to suggestions that impact on axonal traffic is a common mechanism of HSP. In this regard, HSP may be viewed as a central nervous system counterpart of the axonal form of Charcot-Marie-Tooth (CMT2) neuropathy, a clinically and genetically heterogeneous group of disorders of the peripheral nervous system marked by length-dependent distal axon degeneration of motor and sensory nerves [5].

We report on a novel association of an autosomal dominant HSP phenotype with a heterozygous *DNM2* mutation. Dynamins are highly conserved large GTPases that play a critical role in clathrin-mediated endocytosis. They participate in converting the nascent invaginated clathrin-coated pit into a fully formed vesicle and in detaching the vesicle from the plasma membrane (membrane fission) [6]. In developing neurons, both endocytosis and exocytosis are critical for delivery of nutrients and building materials. Clathrin-mediated endocytosis plays a particularly important and specialized role at neuronal synapses [7]. Dynamin isoforms dynamin 1 and dynamin 3 are expressed in neurons, while dynamin 2 is found ubiquitously, including the CNS [8]. Dynamin 2 is involved in several other cellular processes, such as regulation of neuronal morphology, axonal growth and formation of growth cones, centrosome cohesion, actin- and microtubular dynamics [9].

**Fig. 1:**
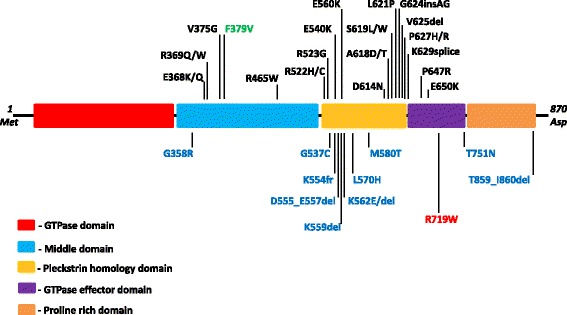
Domain structure of dynamin 2 and location of known disease-causing mutations. Mutations associated with Central nuclear myopathy are shown in black in the upper set; mutations associated with subtypes of Charcot-Marie-Tooth neuropathy are shown in blue at the bottom of the diagram. A homozygous mutation associated with Lethal Congenital Contracture Syndrome appears in green in the upper set. The p.R719W mutation identified in the Siberian family with Hereditary Spastic Paraplegia is shown in red within the bottom set

Dynamin 2 is a 100 kDa multidomain protein composed of a highly conserved catalytic N-terminal GTPase domain (GTPase), a middle domain (MD) driving dynamin oligomerization, a pleckstrin homology domain (PH) critical for the interaction with membrane phosphoinositides, a GTPase effector domain (GED) that activates GTPase upon assembly of dynamin oligomers into higher order structures, and a less conserved C-terminal proline/arginine rich domain (PRD) which directs dynamin to endocytic sites and is a major site for interacting with other proteins [10, 11] (Fig. 1).

Distinct mutations in dynamin 2 have previously been associated with other phenotypes including two forms of Charcot-Marie-Tooth disease: axonal CMT2M (MIM# 606482) originally reported by Züchner et al. [12] and intermediate form CMTDIB (MIM# 606482) reported by Kennerson et al. [13]; centronuclear myopathy ADCNM (MIM# 160150) originally reported by Bitoun et al. [14], and lethal congenital contractures syndrome type 5 LCCS5 (MIM#615368). The majority of CMT-associated *DNM2* mutations is located in the N-terminal part of the pleckstrin homology domain (Fig. 1), while a distinct set of mutations, mainly those found in the middle, PH, and PH/GTPase-effector boundary, is linked to autosomal-dominant centronuclear myopathy. The mechanisms by which various *DNM2* mutations affect discrete tissues and lead to distinct phenotypes remain unknown.

## Methods

### Family pedigree and patient evaluation

Chronic neurological disorders are highly prevalent in the Sakha (Yakut) Republic, Russian Federation. In the process of systematic ascertainment of patients with Viliuisk encephalomyelitis and related disorders [15], a multigenerational HSP family was identified. This 4-generational Siberian family included 9 patients. The oldest family member (I:2, Fig. 2) known to be affected by history had stiff gait and progressive muscle weakness of the lower extremities for at least 10 years before his death at 63. His 3 sons from two marriages (II:1, II:4, and II:6) developed the “family disease”. They were repeatedly examined over the following three decades and diagnosed with hereditary spastic paraplegia. In the third generation, three sons of patient II:4, also from separate marriages, and a daughter of patient II:6 developed the same disease (III:2, III:3, III:5, and III:7). Finally, a great-grandson (IV:1) was the youngest patient to be diagnosed. Pedigree was constructed based on cross interviews of patients and closest family members.

**Fig. 2:**
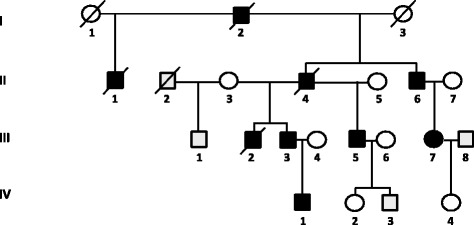
Pedigree of the Siberian family with Hereditary spastic paraplegia. Filled symbols indicate individuals affected with HSP; open symbols represent unaffected family members. The p.R719W mutation was identified in the affected family members II:6, III:2, III:3, III:7, IV:1, but the test for the mutation was negative in unaffected individuals III:1, IV:2, IV:3, IV:4

The study was approved by the Institutional Review Boards of the Institute of Health, North-Eastern Federal University, Yakutsk, and the Yakutsk Research Center, Siberian Branch of the Russian Academy of Medical Sciences. The statement confirmed in writing that the Clinical protocol complied with the Declaration of Helsinki. A written informed consent was obtained from each participant.

After obtaining informed consent, 5 affected and 5 unaffected family members underwent a neurological exam that included assessment of mental status, cranial nerves, muscle strength (MRC scale), coordination, tendon reflexes, muscle bulk, muscle tone, plantar responses, foot deformity, and gait. Evaluation of sensory impairment included clinical testing for pain and temperature sensation, vibration and position sense. Electrophysiological investigation performed in 3 patients (III:3, III:5, and IV:1) included motor and sensory nerve conduction velocities (NCV), compound muscle action potential (CMAP) amplitudes, distal motor latencies (DML), sensory NCV and sensory nerve action potential (SNAP) amplitudes recorded under standard conditions from the median, ulnar, peroneal, tibial, and sural nerves. Routine clinical MRI of the spinal cord was also obtained in three cases. Blood for DNA extraction was drawn from 9 family members.

### Exome sequencing

WES was performed using genomic DNA extracted from peripheral white blood cells of patients II:6 and III:3. Exome capture utilized TruSeq Kit v1 (Illumina, Sand Diego, California) in accordance with manufacturer’s instructions. Library construction, sequence generation, sequence alignment to the reference genome (UCSC GRCh37/hg19), variant calling, and identification of potentially pathogenic variants were performed as described [16]. Variant analysis was performed using an autosomal dominant genetic model. Only variants satisfying stringent coverage and genotype quality criteria were included in further downstream variant analysis. WES generated variants were further validated by segregation analysis to 3 additional affected and 4 unaffected members of the Siberian family using standard Sanger sequencing of amplified DNA fragments.

### Endocytosis assay

The wild-type human *DNM2* in pmCherry-N1 vector (Addgene plasmid 27689) was a kind gift from Dr. C. Merrifield [17]. The c.2155C > T, p.R719W mutation was introduced into the pmCherry-N1 *DNM2* plasmid using the Quick-Change Site Directed Mutagenesis kit according to manufacturer’s protocol (Agilent Technologies, Santa Clara, CA). The integrity of all plasmids was confirmed by DNA sequencing.

HeLa cells were grown in Dulbecco's modified Eagle’s medium (Life Technologies, Grand Island, NY) supplemented with 10 % fetal bovine serum. 50-60 % confluent cells were transfected with plasmids containing wild type or mutant *DNM2* by using transfection reagent HilyMax (Dojindo Molecular Technologies, Rockville, MD) according to the manufacturer’s protocol. Cultures were then incubated in growth medium. At 20 hrs post-transfection, the cells were serum starved for 1 h and treated with 25ug/ml Alexa-Flour 488 conjugated Transferrin (Life Technologies, Frederick, MD) for 15 min at 37 °C. Cells labeled with fluorescent transferrin were then washed and fixed with 4 % paraformaldehyde. Immunofluorescene analysis was performed as previously described [18]. Cells were visualized with a Zeiss LSM 510 confocal microscope. Transferrin immunofluorescent signal was measured by utilizing ImageJ (Image Processing and Analysis in Java, National Institutes of Health) software. Mean fluorescence was first measured in the background; the corrected total transferrin fluorescence signal was then compared between cells expressing WT and mutant DNM2 constructs. Statistical significance was evaluated using the Student’s t-test. P-value <0.05 was considered significant.

### Structural studies

Molecular models of dynamin 2 wild type and p.R719W were generated by I-TASSER [19–21], using dynamin 1 as a template [22]. The sequence homology between dynamin 1 and 2 is 78 % identical overall and 87 % in the GTPase domain. The position of R719 in dynamin 1 is R725. The molecular models were visualized and H-bonds calculated by Chimera [23]. The dynamin 1 tetramer image was generated from docked crystal structures in the 3D density map of K44A-dynamin 1 [24].

## Results

### Phenotypic features and disease outcomes

The pattern of disease inheritance in this family was autosomal dominant (Fig. 2). Clinical information obtained at evaluation of 5 affected family members (II:6, III:3, III:5, III:7, and IV:1) is summarized in Table 1. The illness had an insidious onset between the ages of 10 and 37 years (mean, 26 years) with impaired gait, muscle stiffness and weakness in the lower limbs. Further progression of illness in patients II:6 and III:5 led to severe bilateral lower limb muscle weakness requiring the use of cane and eventual wheel-chair dependency by the 28^th^ and 23^rd^ years of illness. Three patients (I:2, II:1, and III:2) died after an illness lasting for 23 to 32 years, and one (II:4) died in an accident.

**Table 1:** Clinical manifestations of Hereditary spastic paraplegia in members of the Siberian family

Patient	II:6	III:3	III:5	III:7	IV:1
Age at Onset, yrs	29	26	27	37	10
Age at Exam, yrs	62	43	59	39	17
Presenting feature	Weakness spasticity	Weakness spasticity	Muscle weakness	Muscle weakness	Muscle weakness
Cognitive function	normal	forgetful	Cognitive delay (mild)	normal	normal
Bulbar dysfunctions	no	no	dysarthria	no	no
Leg Spasticity	++++	++++	++++	++	+++
Muscle contracture	-	-	+++	-	-
Muscle weakness (MRC scale):					
Knee flexion	3.5	2.5	2	2	4
Knee extention	3.5	3	2	2	4.5
Ankle flexion	4	3	1.5	2.5	5
Ankle extention	3.5	3.5	1.5	2	5
Toes	3	3	1	1.5	3
Muscle wasting	++	++	++	-	-
Hyperactive reflexes:					
Knee	+++	++	++	++	++
Ankle	++	+++	+++	++	+++
Clonus	-	+	+	+	+
Babinski sign	bilateral	no	bilateral	bilateral	bilateral
Decreased abdominal reflexes	no	no	++	no	no
Spastic gait	++++	++++	++++	++	+++
Pinprick	-	-	-	-	-
Vibration	+?	+	+	-	-
Bladder Control	Urinary urgency	normal	Urinary urgency	normal	normal
Pes cavus	+	+	+++	+	++
Ulcerations on legs	++	no	no	no	no
Outcome	Bedridden	Ambulatory	Wheelchair	Ambulatory	Ambulatory

Footnote: "-" or "no" not observed, "+" sign expressed weakly, "++" and "+++" expressed moderately, "++++" expressed strongly, "+?" expressed intermittently, numbers - muscle strength assessment according to the Medical Research Council (MRC) grading scale

At examination (Table 1), a single patient had mild developmental cognitive delay. No optic nerve atrophy, nystagmus, or signs of ataxia were found. Cranial nerves were intact, bulbar functions preserved until late in the illness, none showed dysphagia, and only a single patient (III:5) had moderate spastic dysarthria. Upper extremities muscle bulk, strength, muscle tone, reflexes, sensation and coordination were not affected. In the lower limbs, typical features of upper motor neuron involvement were present in all patients, including spasticity, reduced muscle strength, tightness of Achilles heels. Deep tendon reflexes were increased in all patients with clonus in 3 and bilateral Babinski sign in 4. Moderate muscle atrophy of lower leg muscles was noticed in advanced illness in patients II:6, III:3, III:5, and one patient had flexion contracture of leg muscles and chronic decubitus ulceration (II:6). All patients had profoundly spastic gait. Sphincter control abnormalities manifesting as urinary urgency were observed late in the illness in two patients (II:6 and III:5). Clinical testing for pain, temperature, and position sense did not reveal any deficits. Mild distal vibratory sense impairment was detected in patients II:6 (inconsistently), III:3, and III:5 late in the course of illness. Scoliosis was present in one patient and bilateral *pes cavus* in all examined patients. MRI of the spinal cord performed in patients III:3, III:5, and IV:1 showed no evidence for cord compression, cord atrophy or intrinsic spinal cord abnormalities. There was no evidence of multisystem involvement as determined by routine blood count and routine blood chemistry. In summary, the clinical course in five affected individuals over a multi-decade observation period was overwhelmingly consistent with upper motor neuron dysfunction leading to progressive spasticity. Only late in the course of illness symptoms suggestive of a mild peripheral involvement in the form of mild sensory changes and distal muscle atrophy became apparent. No chronic neurological diseases were found among other members of the extended family or other inhabitants of this village.

### Electrodiagnostic studies

Motor and sensory nerve conduction studies were performed in patients III:3, III:5, and IV:1 (Table 2). Patients III:3 and III:5 were in advanced phases of illness at the 17^th^ and 32^nd^ year from disease onset, patient IV:1 was the youngest in this family at the age of 17 years, in the 7^th^ year after the disease onset. In the n.medianus, conduction studies revealed mostly preserved distal motor latency (DML), motor nerve conduction velocities (NCV) and compound muscle action potentials (CMAP). In the lower limbs, stimulation of n.peroneus profundus and n.tibialis posterior showed reduced NCV and CMAP, most significantly in patients with advanced disease. Sensory nerve action potential (SNAP) amplitudes in n.suralis were also severely affected in patients with advanced disease. The electrodiagnostic study revealed an axonal impairment in motor and sensory peripheral nerves confined to the distal lower extremities .

**Table 2:** Results of nerve conduction studies in patients with Hereditary spastic paraplegia caused by a c.2155C > T, p.R719W mutation in *DNM2*

Patients	III:3	III:5	IV:1	Normal values
Symptom duration (years) at time of study	17	32	7	
Motor nerve conduction studies				
*n.Medianus*				
Distal motor latency (m/s)	3.6	ND	3.5	<4.2
Compound muscle action potential (mV)^a^	**4.5**	ND	8.7	>5.0
Motor nerve conduction velocity (m/s)	48.2	ND	57.1	>47.6
*n.Peroneus*				
Distal motor latency (m/s)	4.49	NR	5.1	<6.5
Compound muscle action potential (mV)^a^	NR	**0.04**	**0.21**	>3.0
Motor nerve conduction velocity (m/s)	NR	NR	40.6	>40.5
*n.Tibialis posterior*				
Distal motor latency (m/s)	NR	NR	**6.2**	<6.0
Compound muscle action potential (mV)^a^	NR	**0.5**	**1.9**	>5.0
Motor nerve conduction velocity (m/s)	NR	NR	39.7	>40.0
Sensory nerve conduction studies				
*n.Medianus*				
Distal sensory nerve conduction velocity (m/s)^b^	61.4	ND	64.5	>47.2
Sensory nerve action potential amplitude (μV)	10	ND	16.6	>5.0
*n.Suralis*				
Distal sensory nerve conduction velocity (m/s)	NR	38.5	42.8	>33.2
Sensory nerve action potential amplitude (μV)	**0.7**	**0.43**	5.82	>6.0

*ND* not done

Bold: outside normal range

^a^Distal motor response amplitude

^b^Orthodromic stimulation

*NR* no response

### Genetic analysis

Prior to exome sequencing, coding regions of common dominant HSP genes had been analyzed by Sanger sequencing, including *SPAST* (spastin) linked to autosomal dominant SPG4 (MIM# 18601) and *ATL1* (atlastin) causing SPG3A (MIM# 182600), genes that account for 37-46 % and 6-11 % of dominant HSP cases [25]. No pathogenic changes were identified.

Exome sequencing was performed in two affected individuals, II:6 and III:3 (Fig. 2). A number of filtering steps were used to prioritize sequence variants (Table 3), beginning with the requirement that the variant had to be shared by two studied affected members of the Siberian family in heterozygous state, assuming a dominant model. Variants in non-coding regions and synonymous SNPs were excluded. Candidates were further restricted by excluding variants reported to have occurred in adult cohort of over 900 subjects participating in the ClinSeq project (ClinSeq *www.genome.gov/20519355*) or the 1000 genome project. Variant population frequency was additionally examined using the ExAC data set. Missense variants were further prioritized by the degree of functional disruption predicted by PolyPhen-II (*www.genetics.bwh.harvard.edu/pph2/**)*, SIFT [26], MutationTaster (*www.mutationtaster.org/*), and CADD (Combined Annotation–Dependent Depletion) - a single-annotation method used to prioritize functional, deleterious and pathogenic variants across many functional categories, effect sizes, and genetic architectures [27]. Exome sequencing did not identify any predicted or known pathogenic changes in previously identified HSP-associated genes. Selected variants were then assessed based on encoded protein expression and function. Considering that our patients had an adult onset neurodegenerative disorder with high penetrance, we excluded genes with no expression in the CNS or genes involved in early embryonic development. Based on analysis of whole exome sequencing data, 4 heterozygous variants were identified under autosomal dominant model. Table 4 provides information regarding the encoded proteins function, expression, and the known disease associations. These four strongest candidates were tested for segregation within the Siberian family (Table 5). The results of this analysis indicated that *DNM2* p.R719W variant is the only one that segregated perfectly.

**Table 3:** Number of genetic variants selected from the overall number of candidates based on each step of the adopted filtering strategy

Filtering steps	Number of variants
HET variants shared by patients II:6 and III:3	51,141
Coding variants	3,664
Variants not present in ClinSeq or 1 K-genome projects	54
Mammalian conserved, PolyPhen-II not benign and/or SIFT not tolerated	28
CADD > =10	13
Selected based on excess HET alleles in ExAC dataset	8
Variants in genes moderately or strongly expressed in the CNS	7
Variants in genes functionally involved in other CNS functions than embryonic development or tumor suppression	4

**Table 4:** Candidate genetic variants identified in the Siberian HSP family based on analysis of whole exome sequencing data

Gene symbol	Encoded protein	Amino acid change	Pathogenic prediction/score			Encoded protein expression	Encoded protein function and known disease associations
			Pphen/SIFT	CADD	Grant-ham		
DLGAP2	Disk large associated protein 2	p.D758N	Damaging/deleterious	24.2	23	Brain	Involved in synaptic function and neuronal cell signaling
DSCAML1	Down syndrome cell adhesion molecule like 1	p.I1742N	Damaging/deleterious	31	149	Brain, heart, liver, pancreas, skeletal muscle	Involved in neuronal and axonal migration, cell adhesion, neuronal self-avoidance
DNAH10	Dynein, axonemal, heavy chain 10	p.V3539M	Damaging/deleterious	17.13	21	Brain, testis, trachea	Involved in axonal transport – moving vesicles, organelles, and signaling molecules along the axon
DNM2	Dynamin 2	p.R719W	Damaging/deleterious	14.7	101	Ubiquitously expressed	Clathrin mediated endocytosis and intracellular membrane trafficking. Linked to Centronuclear myopathy and Charcot-Marie-Tooth neuropathy

**Table 5:** Segregation analysis of four candidate variants in the Siberian HSP family

ID (Fig. 2)	Phenotype	Gene symbol/Variant			
		DLGAP2/p.D758N	DSCAML1/p.I1742N	DNAH10/p.V3539M	DNM2/p.R719W
II:6	Affected	mut	mut	mut	mut
III:2	Affected	mut	ref	ref	mut
III:3	Affected	mut	mut	mut	mut
III:7	Affected	mut	mut	mut	mut
IV:1	Affected	nd	nd	nd	mut
III:1	Unaffected	mut	ref	ref	ref
IV:2	Unaffected	mut	ref	ref	ref
IV:3	Unaffected	ref	nd	ref	ref
IV:4	Unaffected	ref	nd	ref	ref

*mut* mutated allele, *ref* reference allele, *nd* not done. The current age of the unaffected family members (years): III:1 – 57; IV:2 – 39; IV:3 – 27; IV:4 – 30

### *DNM2* variant

**Fig. 3:**
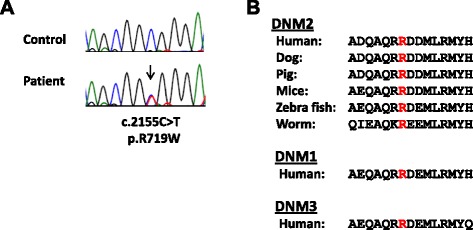
Identification of the disease-causing genetic variant. **a** Sequence chromatograph of a fragment of the *DNM2* gene showing the position of nucleotide substitution (arrow) responsible for the p.R719W mutation. **b** Protein alignment of the GTPase effector domain in various dynamins. Mutated residue is colored red

The identified *DNM2* missense substitution is located at NM_001005360:c.2155C > T; Chr19(GRCh37):g.10939808C > T in exon 19 of the *DNM2* gene, which replaces Arginine (R) with Tryptophan (W) (p.R719W) in the encoded dynamin 2 (Fig. 3a). The region is highly conserved at the amino acid level, up to the worm (Fig. 3b). The p.R719W mutation is in the GTPase effector (GED) domain (Fig. 1). The variant is predicted to be damaging or deleterious by all methods employed in the study. The variant was seen only once in an individual of South Asian descent in the entire Exome Aggregation Consortium data set that comprises exomes of 60,706 unrelated individuals sequenced as part of various disease-specific and population genetic studies (www.exac.broadinstitute.org).

Validation by Sanger sequencing and further segregation to other family members shows that only the affected members II:6, III:2, III:3, III:7, and IV:1 are heterozygous for the c.2155C > T, p.R719W change whereas unaffected individuals III:1, IV:2, IV:3, and IV:4 are homozygous reference (Table 5). We conclude that the *DNM2* c.2155C > T, p.R719W variant is the underlying molecular basis for the HSP phenotype in the Siberian family.

### Functional study

**Fig. 4:**
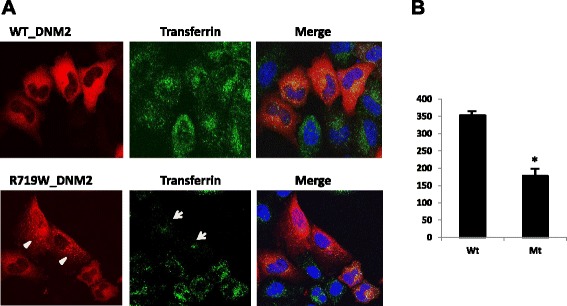
Results of functional analysis of the c.2155C > T, p.R719W mutation. **a** HeLa cells transiently transfected with vectors containing the wild-type (upper panel) and mutant (bottom panel) *DNM2*. The p.R719W mutant exhibits punctate pattern of *DNM2* expression (arrowheads), whereas cells transfected with wild type *DNM2* show diffuse staining of the cytoplasm. Uptake of transferrin is reduced in cells expressing mutant *DNM2* (arrows). Transferrin is labeled by Alexa-Flour 488 (green); nuclei are labeled with blue stain. **b** Quantification of transferrin uptake. The histogram represents the mean +/− standard error of the corrected total transferrin fluorescence of WT (n = 30 cells) and p.R719W (n = 25 cells); P < 0.001

To investigate the effects of the disease-related dynamin 2 p.R719W mutant, we evaluated clathrin-mediated endocytosis in HeLa cells transiently transfected with the wild type and mutant dynamin 2 following incubation with fluorescently labeled transferrin. We observed a prominent punctate staining in cells transfected with p.R719W *DNM2* (Fig. 4), a phenomenon morphologically similar to that reported for other *DNM2* mutations causing CMT and CNM [28]. There was a significant decrease in transferrin uptake in cells expressing mutant dynamin 2. This decrease was evident in all transfected cells and was especially significant (more than 50 %) in cells marked by arrows. The granules are localized most prominently in the perinuclear area, likely in the endosomal compartment. The results suggest that inhibited endocytosis is part of the pathophysiological mechanisms leading to HSP in this family.

### Structural models

**Fig. 5:**
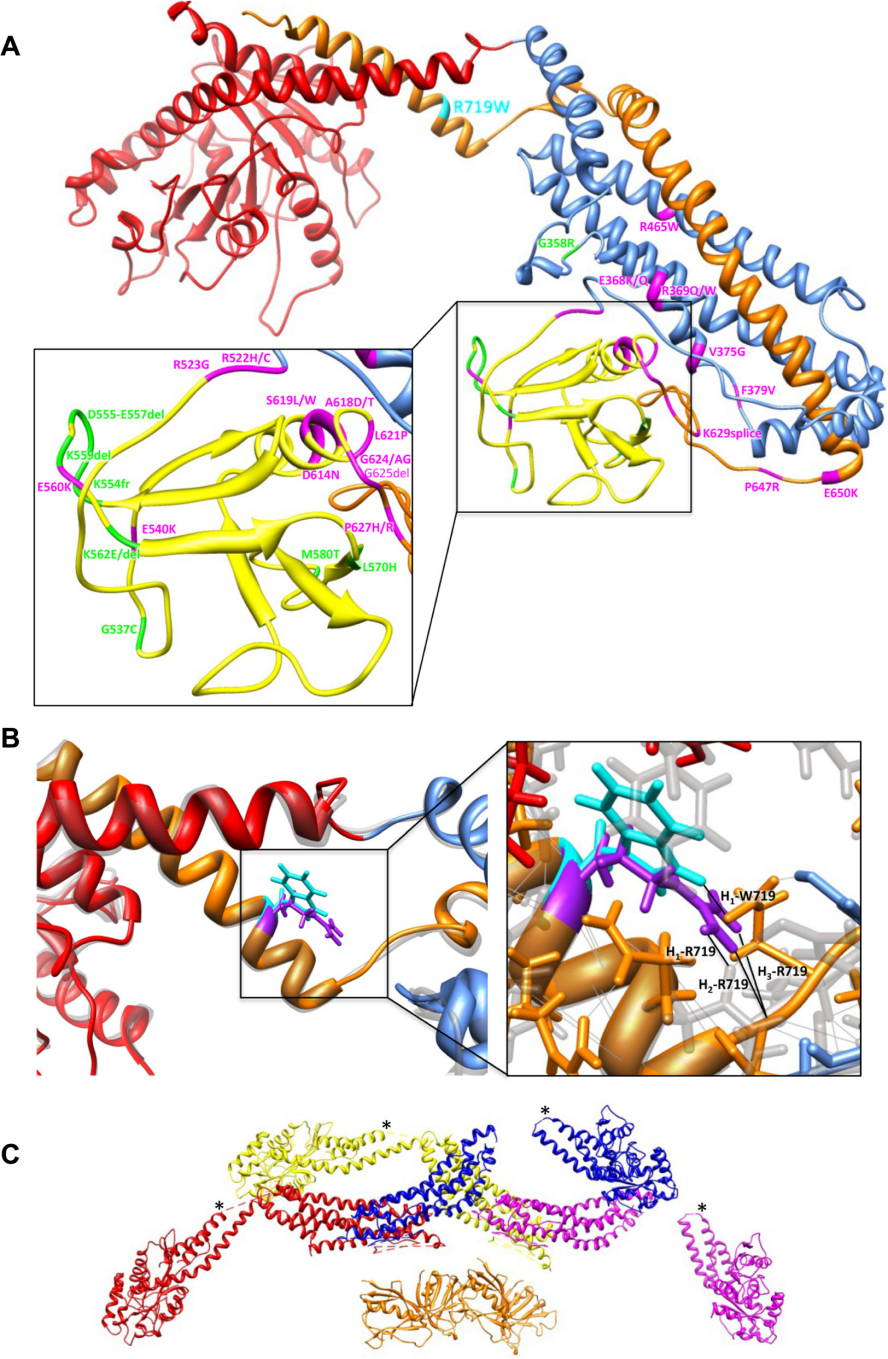
Structural modeling of dynamin 2 c.2155C > T, p.R719W mutation. **a** Molecular model of dynamin 2 based on the crystal structure of dynamin 1 [22] indicating the HSP p.R719W mutation (cyan), CNM mutations (magenta) and CMT mutations (green). Dynamin domains are colored as follows: GTPase domain (red), Middle (blue), PH (yellow) and GED (orange). p.R719W is located at the hinge region between the BSE (a three helix bundle consisting of N and C termini of the G domain and the C termini of GED) and the stalk (Middle and GED) of dynamin. Generated by I-TASSER. **b** Overlay of wild-type and p.R719W dynamin 2 molecular models with R719 (purple) and W719 (cyan) side chains shown. On the right panel: there are three putative H bonds connecting R719 to the rest of the molecule (labeled H_1–3_-R719) compared to only one for W719 (H_1_-W719). **c** The assembled tetramer of dynamin 1 was generated from docking crystal structures into a 3D density map of K44A-dynamin 1 [24]. Dynamin monomers are colored red, yellow, blue and purple. Asterisks indicate the location of R725W (equivalent to R719W in dynamin 2) in the assembled dynamin 1

The HSP mutation p.R719W is located in the three-helix bundle of dynamin called the bundle-signaling element (BSE) (Fig. 5a). It is intriguing that p.R719W is the only disease-associated mutation found in this region. Both CMT and CNM mutations are located in the stalk (Middle/GED) and PH domains of the protein (Fig. 5a). The BSE undergoes a large conformational change upon GTP hydrolysis that has been described as the power stroke of dynamin [29, 30]. A mutation in this region suggests a defect in propagating the BSE conformational change to the rest of the molecule and assembled helical polymer. In addition, the mutation is located near a hinge between the BSE and the stalk and as indicated by fewer H-bonds (1 H-bond in W719 compared to 3 H-bonds in R719), p.R719W may disrupt the connection between these domains (Fig. 5b). The potential disruption of R719W in the assembled protein is further illustrated by examining the dynamin tetramer (Fig. 5c, asterisks).

## Discussion

The Siberian family under study had a history compatible with autosomal dominant transmission of a young-adult onset slowly progressive bilateral leg spasticity corresponding to typical clinical manifestations of HSP: each studied patient had lower extremity hyperreflexia, muscle weakness, and spastic gait. Some patients also showed wasting of lower leg muscles, *pes cavus*, decreased vibratory sense in the ankles, and urinary urgency as late features of illness. There was no optic neuropathy, retinopathy, extrapyramidal symptoms, dementia, ataxia, ichthyosis, or deafness described as part of the “complicated” subtypes of HSP. Alternative diagnoses were excluded based on the results of clinical studies and routine laboratory investigations. Nerve conduction studies in patients with advanced disease revealed motor and sensory axonal neuropathy in distal lower extremities, a feature reported in other subtypes of HSP [31].

Exome sequencing methodology was used for genetic analysis of this HSP family. There are well known limitations to exome sequencing as a method of detection of the causative mutation. Structural genomic alterations, changes in regions not captured by this technology such as translocations, changes in regulatory regions or introns, may be responsible for the clinical phenotypes. But subsequent analysis of tissue expression, structural and functional characteristics of the candidate genetic variants, and segregation within the pedigree as applied in this study are helpful in the process of detection of the disease-associated variant.

The identification of the *DNM2* p.R719W mutation as the cause of HSP in this family brought attention to the fact that mutations in this same gene are responsible for other phenotypes, including two forms of CMT peripheral neuropathy. *DNM2*-associated autosomal dominant CMT2 neuropathy is characterized by progressive distal muscle weakness and atrophy, predominantly in the lower extremities, decreased or absent tendon reflexes, steppage gait, foot drop, *pes cavus* [32, 33]. These clinical features are in contrast with the presentation observed in patients with *DNM2*-associated HSP described here, although some overlap with CMT2 neuropathy exists, especially late in the disease. There has been active discussion on the subject of similarity between some forms of CMT neuropathy and HSP based on the identification of shared mechanisms involving central and peripheral motor neurons (for review, see [5]). Interestingly, mutations in *KIF5A*, a gene encoding the neuronal kinesin heavy chain implicated in anterograde axonal transport, have been associated with “pure” HSP, although a fraction of family members shows electrophysiological evidence of sensory-motor peripheral axonal neuropathy [34, 35]. Our findings give some weight to the view that HSP and CMT caused by *DNM2* mutations, although clinically and anatomically separate syndromes, may have some overlap due to shared pathological vulnerabilities in their extended axonal processes.

Another *DNM2*-associated disease, autosomal-dominant Centronuclear myopathy, is a slowly progressive congenital myopathy characterized by delayed motor milestones, generalized muscle weakness, ptosis, and ophthalmoplegia [14]. It includes a wide spectrum of phenotypes ranging from severe neonatal to mild late-onset familial forms [36, 37]. CNM is characterized pathologically by myofiber atrophy, abnormal nuclear centralization and internalization, and type 1 muscle fiber predominance [38]. Some patients with CNM show clinical overlap of myopathy with mild axonal involvement in peripheral nerves [39].

It remains to be determined why specific *DNM2* mutations result in involvement of skeletal muscle, peripheral nerves, peripheral or corticospinal neurons. Existing hypotheses concentrate on different roles of dynamin 2 domains involved in CMT or CNM. CMT mutations are located within the pleckstrin homology [12] and proline-rich domains [33], whereas the CNM mutations are found within the PH domain. The significance of involvement of specific dynamin domains is further underlined by the evidence we obtained in this study that the HSP dynamin 2 mutation, p.R719W, is the only known mutation in the bundle-signaling element of dynamin, which is structurally and most likely functionally different from the stalk and PH domains in which CMT and CNM mutations are located. A mutation in this region may cause a conformational change to the helical element and affect dynamin assembly. Moreover, the mutation results in fewer hydrogen bonds between the bundle-signaling element and the stalk. A weaker connection potentially uncouples the power stroke of dynamin from the rest of the molecule, a step that is predicted to be essential for endocytosis [30].

Modeling in cell cultures of all known *DNM2* mutations causing either disease show their destructive effect on clathrin-mediated endocytosis considered a major mechanism for uptaking molecules into eukaryotic cells regulated by dynamin [40, 41]. Clathrin-coated pits capture cargo molecules via adaptors, the pits invaginate and pinch off to form clathrin-coated vesicles that carry the cargo into the cell. Data presented here show that the HSP-causing dynamin 2 p.R719W mutation induces a marked decrease of clathrin-mediated endocytosis similarly to other disease-causing dynamin 2 mutations, suggesting that impairment of the membrane trafficking process contributes to the pathogenesis of all *DNM2*-associated diseases. Prominent punctate staining in mutant cells also suggests that the HSP *DNM2* mutation affects cellular morphology by mechanisms similar to previously characterized CMT and CNM mutations [12, 28].

The superfamily of dynamin GTPases comprises a group of proteins that are involved in diverse cellular functions and are often closely associated with biological membranes; they function in vesicle scission, as well as in fusion and fission of organelles [9]. The large GTPase atlastin belongs to the dynamin superfamily and shares the domain architecture with dynamin 2. Atlastin is involved in ER fusion, vesicular trafficking, axon formation and elongation. Mutations in atlastin isoform 1 (atlastin 1) have been identified in patients with early-onset HSP (SPG3). Curiously, mutations in atlastin 1 have also been associated with hereditary sensory neuropathy type 1D (HSN1D; MIM# 613708), a condition characterized by distal axonal sensory deficits leading to late distal skin ulceration and amputation, with some patients showing upper motor involvement [42]. The majority of atlastin 1 mutations are located in the GTPase domain, although some have been identified in the stalk, the BSE, as well as in the transmembrane domains [43] suggesting that membrane association that is common to both representatives of the dynamin family, dynamin 2 and atlastin 1, may be critical in the pathophysiology of HSP.

Lack of *DNM2* mutations in other Siberian HSP families suggests that more than a single gene is responsible for this phenotype; HSP is an extremely heterogeneous disorder with mutations in more than 60 genes associated with this syndrome.

## Conclusions

Patients in the 4-generation Siberian family suffered from hereditary spastic paraplegia and mild peripheral axonopathy. Our molecular, structural and functional analyses and segregation studies allowed to identify *DNM2* c.2155C > T, p.R719W mutation as a cause of this disease. The mutation is located in a unique position of the multidomain dynamin 2 protein and its pathogenic effects may depend on structural relationships this mutation disrupts. Awareness of the distinct, but partly overlapping clinical phenotypes associated with mutations in *DNM2,* will facilitate identification of these disorders in additional families and direct future research toward better understanding of the role dynamin 2 and related networks have in health and disease.
